# Supplementary material for: Empanelment of health care facilities under Ayushman Bharat Pradhan Mantri Jan Arogya Yojana (AB PM-JAY) in India
Source: PLoS One. 2021 May 27;16(5):e0251814. doi: 10.1371/journal.pone.0251814 (PMC8158976; doi:10.1371/journal.pone.0251814)
Supplement: S1 Table — (DOCX) [file pone.0251814.s001.docx]

**S1 Table. Distribution of Public Sector PMJAY Empanelment across Levels of Care and by Mode of implementation**

| **Type of Facility** | **Hybrid Mode** | | **Insurance Mode** | | **Trust Mode** | | **NHCP** | | **Total** | |
| --- | --- | --- | --- | --- | --- | --- | --- | --- | --- | --- |
|  | **No of EHCFs** | **Estimated Beds** | **No of EHCFs** | **Estimated Beds** | **No of EHCFs** | **Estimated Beds** | **No of EHCFs** | **Estimated Beds** | **No of EHCFs**  **(% share of total facilities)** | **Estimated Beds**  **(% share of total Beds)** |
| Sub Health Centre (SHC)/ Primary Health Centre (PHC)/ Urban Primary Health Centres (UPHC) | 1430 | 14550 | 140 | 1424 | 3018 | 31119 | 0 | 0 | **4588  (40.4%)** | **47093  (8.4%)** |
| Community Health Centre (CHC)/Urban Community Health Centre (UCHC) | 495 | 14063 | 294 | 7991 | 2524 | 76924 | 0 | 0 | **3313**  **(29.1%)** | **98978**  **(17.6%)** |
| Sub District Hospital (SDH) | 374 | 30950 | 137 | 12632 | 404 | 30524 | 0 | 0 | **915**  **(8.0%)** | **74106**  **(13.2%)** |
| District Hospital (DH) / General Hospital. (GH)/Women and Child Hospitals (W&C) | 244 | 34098 | 119 | 24553 | 593 | 101207 | 4 | 590 | **960**  **(8.4%)** | **160448 (28.5%)** |
| Medical College (MC)/Dental Medical Colleges (MCD) | 706 | 43299 | 29 | 22188 | 94 | 65070 | 5 | 8744 | **834**  **(7.3%)** | **139301 (24.8%)** |
| Others (Specialty Hospitals/Military Hospitals/Railway Hospital/ESI hospitals/PSU Hospitals) | 170 | 8901 | 92 | 5957 | 355 | 21221 | 140 | 6631 | **757**  **(6.7%)** | **42710**  **(7.6%)** |
| **Total** | **3419** | **145861** | **811** | **74745** | **6988** | **326065** | **149** | **15965** | **11367** | **562636** |

Note: For understanding the availability of beds in public health facilities, sanctioned beds were collated from reports available on state health department and National Health Mission (NHM) websites wherever available else the maximum bed strength as per IPHS standard was allocated and bed strength was estimated.
