# Supplementary material for: Empanelment of health care facilities under Ayushman Bharat Pradhan Mantri Jan Arogya Yojana (AB PM-JAY) in India
Source: PLoS One. 2021 May 27;16(5):e0251814. doi: 10.1371/journal.pone.0251814 (PMC8158976; doi:10.1371/journal.pone.0251814)
Supplement: S3 Table — (DOCX) [file pone.0251814.s003.docx]

**S3 Table. State Wise Distribution of Public Health Care Facility Empanelment under PMJAY by Geography**

| **State Name** | **Mode of  Implementation** | **SHC/PHC/ UPHC** | **CHC/UCHC** | **SDH** | **DH/GH/**  **W&C** | **MC/MCD** | **Other** |
| --- | --- | --- | --- | --- | --- | --- | --- |
| **North** | | | | | | | |
| Chandigarh | Trust | 0 | 2 (40%) | 1 (20%) | 0 | 1 (20%) | 1 (20%) |
| NCT of Delhi | NHCP | 0 | 0 | 0 | 1 (3.8%) | 4 (15.4%) | 21 (80.8%) |
| Haryana | Trust | 2 (1.2%) | 90 (54.2%) | 36 (21.7%) | 25 (15.1%) | 4 (2.4%) | 9 (5.4%) |
| Himachal Pradesh | Trust | 18 (12.6%) | 44 (30.8%) | 49 (34.3%) | 25 (17.5%) | 5 (3.5%) | 2 (1.4%) |
| Jammu & Kashmir | Insurance | 1 (0.8%) | 65 (51.6%) | 16 (12.7%) | 30 (23.8%) | 5 (4.0%) | 9 (7.1%) |
| Punjab | Insurance | 0 | 136 (56.4%) | 44 (18.3%) | 21 (8.7%) | 4 (1.7%) | 36 (14.9%) |
| Rajasthan | Trust | 0 | 452 (76%) | 52 (8.7%) | 34 (5.7%) | 6 (1%) | 51 (8.6%) |
| Uttarakhand | Trust | 0 | 64 (52%) | 1 (0.8%) | 34 (27.6%) | 4 (3.3%) | 20 (16.3%) |
| **Central** | | | | | | | |
| Chhattisgarh | Trust | 468 (65.5%) | 155 (21.7%) | 14 (2%) | 23 (3.2%) | 2 (0.3%) | 52 (7.3%) |
| Madhya Pradesh | Trust | 10 (2.4%) | 253 (61.6%) | 45 (10.9%) | 57 (13.9%) | 9 (2.2%) | 37 (9%) |
| Uttar Pradesh | Trust | 22 (2%) | 820 (75%) | 15 (1.4%) | 145 (13.3%) | 22 (2.0%) | 69 (6.3%) |
| **East** | | | | | | | |
| Bihar | Trust | 312 (54.6%) | 166 (29.1%) | 41 (7.2%) | 37 (6.5%) | 10 (1.8%) | 5 (0.9%) |
| Jharkhand | Hybrid | 2 (0.7%) | 176 (64.2%) | 12 (4.4%) | 26 (9.5%) | 4 (1.5%) | 54 (19.7%) |
| Odisha | NHCP | 0 | 0 | 0 | 2 (6.9%) | 1 (3.4%) | 26 (89.7%) |
| West Bengal | NHCP | 0 | 0 | 0 | 0 | 0 | 59 (100%) |
| **Northeast** | | | | | | | |
| Arunachal Pradesh | Trust | 0 | 0 | 0 | 3 (60%) | 1 (20%) | 1 (20%) |
| Assam | Trust | 25 (15.6%) | 82 (51.3%) | 11 (6.9%) | 26 (16.3%) | 7 (4.4%) | 9 (5.6%) |
| Manipur | Trust | 2 (4%) | 4 (8%) | 2 (4.0%) | 7 (14%) | 2 (4%) | 33 (66%) |
| Meghalaya | Insurance | 111 (68.1%) | 27 (16.6%) | 0 | 10 (6.1%) | 2 (1.2%) | 13 (8%) |
| Mizoram | Trust | 57 (66.3%) | 9 (10.5%) | 2 (2.3%) | 9 (10.5%) | 1 (1.2%) | 8 (9.3%) |
| Nagaland | Insurance | 27 (36%) | 20 (26.7%) | 0 | 11 (14.7%) | 0 | 17 (22.7%) |
| Sikkim | Trust | 0 | 0 | 0 | 4 (44.4%) | 0 | 5 (55.6%) |
| Tripura | Trust | 45 (45%) | 22 (22%) | 11 (11%) | 6 (6%) | 3 (3%) | 13 (13%) |
| **West** | | | | | | | |
| Dadra & Nagar Haveli | Insurance | 0 | 2 (50%) | 1 (25.0%) | 1 (25.0%) | 0 | 0 |
| Daman & Diu | Insurance | 0 | 1 (33.3%) | 0 | 2 (66.7%) | 0 | 0 |
| Goa | Trust | 0 | 6 (54.5%) | 1 (9.1%) | 3 (27.3%) | 1 (9.1%) | 0 |
| Gujarat | Hybrid | 1427 (78.5%) | 316 (17.4%) | 1 (0.1%) | 52 (2.9%) | 8 (0.4%) | 13 (0.7%) |
| Maharashtra | Hybrid | 0 | 0 | 25 (20.3%) | 42 (34.1%) | 22 (17.9%) | 34 (27.6%) |
| **South** | | | | | | | |
| Andaman & Nicobar | Trust | 0 | 0 | 0 | 3 (100%) | 0 | 0 |
| Andhra Pradesh | Trust | 0 | 141 (62.7%) | 51 (22.7%) | 23 (10.2%) | 3 (1.3%) | 7 (3.1%) |
| Karnataka | Trust | 2057 (81.7%) | 214 (8.5%) | 72 (2.9%) | 128 (5.1%) | 13 (0.5%) | 33 (1.3%) |
| Kerala | Insurance | 1 (0.5%) | 39 (20.9%) | 76 (40.6%) | 40 (21.4%) | 14 (7.5%) | 17 (9.1%) |
| Lakshadweep | Trust | 0 | 0 | 0 | 1 (100%) | 0 | 0 |
| Puducherry | Insurance | 0 | 4 (33.3%) | 0 | 4 (33.3%) | 4 (33.3%) | 0 |
| Tamil Nadu | Hybrid | 1 (0.1%) | 3 (0.2%) | 336 (27.9%) | 124 (10.3%) | 672 (55.8%) | 69 (5.7%) |
| Telangana | NHCP | 0 | 0 | 0 | 1 (8.3%) | 0 | 11 (91.7%) |
| PSU | NHCP | 0 | 0 | 0 | 0 | 0 | 23 (100%) |
| **Total** |  | **4588** | **3313** | **915** | **960** | **834** | **757** |
