# Supplementary material for: Empanelment of health care facilities under Ayushman Bharat Pradhan Mantri Jan Arogya Yojana (AB PM-JAY) in India
Source: PLoS One. 2021 May 27;16(5):e0251814. doi: 10.1371/journal.pone.0251814 (PMC8158976; doi:10.1371/journal.pone.0251814)
Supplement: S5 Table — (DOCX) [file pone.0251814.s005.docx]

**S5 Table. State Wise Estimates of Number of Beds in Public Empanelled Health Care Facilities under PMJAY by Geography**

| **Row Labels** | **Mode of Implementation** | **SHC/PHC/ UPHC** | **CHC/UCHC** | **SDH** | **DH/GH/**  **W&C** | **MC/MCD** | **Other** | **Total available beds** |
| --- | --- | --- | --- | --- | --- | --- | --- | --- |
| **North** | | | | | | | | |
| Chandigarh | Trust | 0 | 100  (6.7%) | 100 (6.7%) | 0 | 800 (53.3%) | 500 (33.3%) | 1500 |
| NCT of Delhi | NHCP | 0 | 0 | 0 | 380  (4%) | 7990 (84.8%) | 1053 (11.2%) | 9423 |
| Haryana | Trust | 60  (0.5%) | 2291  (18%) | 1959 (15.4%) | 4425 (34.9%) | 3608 (28.4%) | 353  (2.8%) | 12696 |
| Himachal Pradesh | Trust | 430  (4.4%) | 1553 (15.8%) | 3215 (32.7%) | 2954 (30.1%) | 1602 (16.3%) | 67  (0.7%) | 9821 |
| Jammu and Kashmir | Insurance | 22  (0.2%) | 1884 (17.6%) | 525 (4.9%) | 5065 (47.3%) | 1542 (14.4%) | 1660 (15.5%) | 10698 |
| Punjab | Insurance | 0 | 3241 (25.7%) | 3082 (24.4%) | 3033 (24%) | 2561 (20.3%) | 706  (5.6%) | 12623 |
| Rajasthan | Trust | 0 | 13560 (42.4%) | 3190 (10%) | 5632 (17.6%) | 7943 (24.8%) | 1658 (5.2%) | 31983 |
| Uttarakhand | Trust | 0 | 1680 (30.3%) | 100 (1.8%) | 3394 (61.2%) | 0 | 373  (6.7%) | 5547 |
| **Central** | | | | | | | | |
| Chhattisgarh | Trust | 5200 (28.6%) | 4916 (27.1%) | 615 (3.4%) | 4115 (22.6%) | 800 (4.4%) | 1476 (8.1%) | 18168 |
| Madhya Pradesh | Trust | 120  (0.3%) | 7926  (21%) | 3218 (8.5%) | 16575 (43.9%) | 7172 (19%) | 2704 (7.2%) | 37715 |
| Uttar Pradesh | Trust | 278  (0.4%) | 24819 (35.9%) | 2049 (3%) | 19011 (27.5%) | 17601 (25.4%) | 5409 (7.8%) | 69167 |
| **East** | | | | | | | | |
| Bihar | Trust | 3120 (13.3%) | 4967 (21.1%) | 3012 (12.8%) | 4746 (20.2%) | 7232 (30.7%) | 465  (2%) | 23542 |
| Jharkhand | Hybrid | 10  (0.1%) | 4098 (35%) | 451 (3.9%) | 2387 (20.4%) | 2664 (22.8%) | 2098 (17.9%) | 11708 |
| Odisha | NHCP | 0 | 0 | 0 | 200  (8.3%) | 754 (31.3%) | 1457 (60.4%) | 2411 |
| West Bengal | NHCP | 0 | 0 | 0 | 0 | 0 | 3299 (100%) | 3299 |
| **Northeast** | | | | | | | | |
| Arunachal Pradesh | Trust | 0 | 0 | 0 | 283 (53.1%) | 250 (46.9%) | 0 | 533 |
| Assam | Trust | 304  (2.0%) | 2513 (16.5%) | 760  (5%) | 4567 (30.0%) | 6003 (39.5%) | 1065  (7%) | 15212 |
| Manipur | Trust | 13  (0.5%) | 77  (2.7%) | 61 (2.1%) | 586 (20.6%) | 1636 (57.5%) | 470 (16.5%) | 2843 |
| Meghalaya | Insurance | 1110 (23.5%) | 790 (16.7%) | 0 | 1680 (35.6%) | 550 (11.6%) | 595 (12.6%) | 4725 |
| Mizoram | Trust | 582  (28.3%) | 230 (11.2%) | 60 (2.9%) | 773 (37.6%) | 300 (14.6%) | 110  (5.4%) | 2055 |
| Nagaland | Insurance | 272  (13.8%) | 546 (27.8%) | 0 | 920 (46.8%) | 0 | 227 (11.6%) | 1965 |
| Sikkim | Trust | 0 | 0 | 0 | 402 (27.8%) | 0 | 1042 (72.2%) | 1444 |
| Tripura | Trust | 448  (10.9%) | 635 (15.5%) | 795 (19.4%) | 750 (18.3%) | 1205 (29.4%) | 260  (6.4%) | 4093 |
| **West** | | | | | | | | |
| Dadra and Nagar Haveli | Insurance | 0 | 60  (12.6%) | 100 (21%) | 316 (66.4%) | 0 | 0 | 476 |
| Daman and Diu | Insurance | 0 | 42  (16.3%) | 0 | 215 (83.7%) | 0 | 0 | 257 |
| Goa | Trust | 0 | 240 (11.2%) | 75 (3.5%) | 684 (31.8%) | 1152 (53.6%) | 0 | 2151 |
| Gujarat | Hybrid | 14510 (34.8%) | 9875 (23.7%) | 75 (0.2%) | 13538 (32.5%) | 1663 (4%) | 2055 (4.9%) | 41716 |
| Maharashtra | Hybrid | 0 | 0 | 3930 (11.2%) | 13021 (37.2%) | 15471 (44.2%) | 2550 (7.3%) | 34972 |
| **Row Labels** | **Mode of Implementation** | **SHC/PHC/ UPHC** | **CHC/UCHC** | **SDH** | **DH/GH/**  **W&C** | **MC/MCD** | **Other** | **Total available beds** |
| **South** | | | | | | | | |
| Andaman and Nicobar | Trust | 0 | 0 | 0 | 666 (100%) | 0 | 0 | 666 |
| Andhra Pradesh | Trust | 0 | 4500  (18%) | 4860 (19.5%) | 11817 (47.3%) | 2012 (8.1%) | 1786 (7.2%) | 24975 |
| Karnataka | Trust | 20564 (32.7%) | 6917 (11%) | 6455 (10.3%) | 19777 (31.4%) | 5754 (9.1%) | 3483 (5.5%) | 62950 |
| Kerala | Insurance | 20  (0.1%) | 1308 (3.3%) | 8925 (22.6%) | 12097 (30.7%) | 14345 (36.3%) | 2769 (7.0%) | 39464 |
| Puducherry | Insurance | 0 | 120  (2.6%) | 0 | 1227 (27%) | 3190 (70.3%) | 0 | 4537 |
| Lakshadweep | Trust | 0 | 0 | 0 | 50  (100%) | 0 | 0 | 50 |
| Tamil Nadu | Hybrid | 30  (0.1%) | 90  (0.2%) | 26494 (46.1%) | 5152  (9%) | 23501 (40.9%) | 2198 (3.8%) | 57465 |
| Telangana | NHCP | 0 | 0 | 0 | 10  (1.7%) | 0 | 570 (98.3%) | 580 |
| PSU | NHCP | 0 | 0 | 0 | 0 | 0 | 252 (100%) | 252 |
| **Total** |  | **47093** | **98978** | **74106** | **160448** | **139301** | **42710** | **563682** |
