# Supplementary material for: Empanelment of health care facilities under Ayushman Bharat Pradhan Mantri Jan Arogya Yojana (AB PM-JAY) in India
Source: PLoS One. 2021 May 27;16(5):e0251814. doi: 10.1371/journal.pone.0251814 (PMC8158976; doi:10.1371/journal.pone.0251814)
Supplement: S6 Table — (DOCX) [file pone.0251814.s006.docx]

**S6 Table State wise availability of Public Empanelled Hospitals per Hundred Thousand eligible population by mode of implementation**

| **State Name** | **Total Beds Available in Empanelled Public Sector Hospitals** | **Beds per 100,000 eligible population** |
| --- | --- | --- |
| Hybrid | | |
| Gujarat | 41716 | 109 |
| Jharkhand | 11708 | 37 |
| Maharashtra | 34972 | 90 |
| Tamil Nadu | 57465 | 99 |
| Insurance | | |
| Dadra and Nagar Haveli | 476 | 74 |
| Daman and Diu | 257 |  |
| Jammu and Kashmir | 10698 | 316 |
| Kerala | 39464 | 223 |
| Meghalaya | 4725 | 114 |
| Nagaland | 1965 | 164 |
| Puducherry | 4537 | 1062 |
| Punjab | 12623 | 53 |
| Trust | | |
| Andhra Pradesh | 24975 | 45 |
| Andaman and Nicobar | 666 | 770 |
| Arunachal Pradesh | 533 | 39 |
| Assam | 15212 | 114 |
| Bihar | 23542 | 39 |
| Chandigarh | 1500 | 499 |
| Chhattisgarh | 1948 | 8 |
| Goa | 2151 | 1359 |
| Haryana | 12696 | 152 |
| Himachal Pradesh | 9821 | 436 |
| Karnataka | 62950 | 115 |
| Lakshadweep | 50 | 582 |
| Madhya Pradesh | 37715 | 70 |
| Manipur | 2843 | 195 |
| Mizoram | 2055 | 223 |
| Rajasthan | 31983 | 55 |
| Sikkim | 1444 | 780 |
| Tripura | 4093 | 193 |
| Uttarakhand | 5547 | 72 |
| Uttar Pradesh | 69167 | 87 |
